# MicroRNA-134 plasma levels before and after treatment with valproic acid for epilepsy patients

**DOI:** 10.18632/oncotarget.20292

**Published:** 2017-08-16

**Authors:** Xiaofeng Wang, Yifeng Luo, Shuangxi Liu, Liming Tan, Sanhu Wang, Rongyong Man

**Affiliations:** ^1^ Department of Neurology, The Third Affiliated Hospital of Southern Medical University, Guangzhou, 510630 China; ^2^ Department of Neurology of the First People's Hospital of Huaihua, affiliated to University of South China, Huaihua, Hunan 418000, China; ^3^ Department of Pharmacy of the First People's Hospital of Huaihua, affiliated to University of South China, Huaihua, Hunan 418000, China; ^4^ Medical Research Center of the First People's Hospital of Huaihua, affiliated to University of South China, Huaihua, Hunan 418000, China

**Keywords:** microRNA-134, valproic acid, epilepsy, biomarker, temporal lobe epilepsy

## Abstract

**Background:**

Temporal lobe epilepsy is the second most common neurological disorders characterized by recurrent spontaneous seizures. MicroRNAs play a vital role in regulating synaptic plasticity, brain development and post-transcriptional expression of proteins. In both animal models of epilepsy and human patients, miR-134, a brain-specific microRNA has recently been identified as a potential regulator of epileptogenesis.

**Methods:**

microRNA identified as targets for the actions of valproic acid (VPA) are known to have important effects in brain function. In this study, 59 new-onset epilepsy patients and 20 controls matched by sex and age were enrolled. Patients with a score < 3 were allocated into the mild group, 3-5 into the moderate group and >5 into the severe group. The plasma miRNA-134 level was quantitatively measured using real-time PCR.

**Results:**

Plasma miRNA-134 level in new-onset epilepsy patients was significantly up-regulated when compared with that in healthy controls, and then considerably down-regulated after oral intake of valproic acid medication. The up-regulated plasma miRNA-134 levels may be directly associated with the pathophysiology and severity of epilepsy.

**Conclusion:**

Plasma miRNA-134 in epilepsy may be considered as a potential peripheral biomarker that responds to the incidence of epilepsy and associates with use of anti-epilepsy drugs.

## INTRODUCTION

Epilepsy is one of the most disabling medical disorders that affect a total of 65 million patients’ survival and quality of life globally. Although genetic and familial studies strongly suggest that a neurobiological basis may underlie the pathophysiology of epilepsy, its etiology remains poorly understood [1]. In recent years, there is a growing awareness that epilepsy can best be conceptualized as genetically influenced disorders of synapses and circuits, rather than simply as deficits or excesses in individual neurotransmitters [2]. In this context, microRNAs (miRNAs) have been proven to affect the function of multiple organs, such as brain development, dendritic spine morphology and epilepsy, *etc*. [3–5]. Moreover, miRNA identified as targets for the actions of chronic VPA are known to play an intriguing role in brain function [6]. The prime example of a miRNA, which is involved in remodeling of neuronal structures in consequence of epilepsy, is miRNA-134 [7]. This miRNA regulates the size of dendritic spines and has been involved in the control of neuronal microstructure [8]. MiRNA-134 represses the translation of the LIM kinase-1 (LIMK1) mRNA, a protein kinase that phosphorylates cofilin and inactivates the ability of cofilin to depolymerize actin, and loss of Limk1 results in abnormal spine morphology. It is reported that miRNA-134 is up-regulated in experimental epilepsy and silencing miRNA-134 generates a seizure-refractory state and attenuates epileptic seizures and the pathophysiological features of temporal lobe epilepsy (TLE).

Considering the function of miRNA-134 in epilepsy, we hypothesize that miRNA-134 regulates distinct characteristics of the severity of epilepsy. Furthermore, circulating miRNAs from the serum or plasma of patients have been shown to be stable predictors of epilepsy diseases [9–11]. In this study, the changes of plasma miRNA-134 levels during epileptic seizure were investigated to evaluate the effect of valproate acid drugs upon the miRNA-134 levels.

## RESULTS

No statistical significance was identified between epilepsy patients and the healthy controls regarding gender distribution, age (28.23±12.37 years for healthy controls and 29.82±9.24 years for epilepsy patients; *P*=0.940) and the number of seizure monthly among different groups (all *P* >0.05).

**Figure 1:**
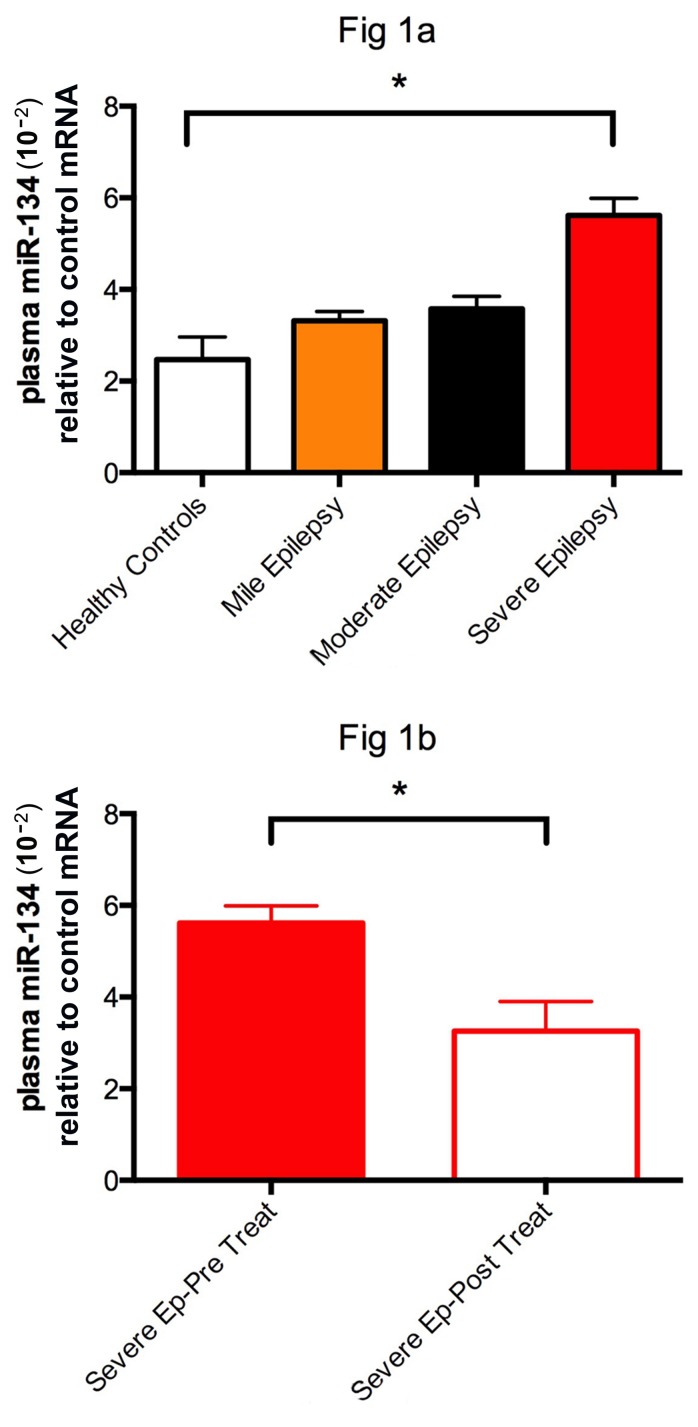
The relative expression of miRNA-134 in patients with new-onset severe epilepsy was (5.62±1.60), significantly higher than (2.47±1.90) of the healthy controls * represent significant fold change between two groups.

As illustrated in Figure 1a, the relative expression level of miRNA-134 in the patients with new-onset severe epilepsy was (5.62±1.60), significantly higher than that of the healthy controls (2.47±1.90). However, no significant difference was identified between the patients with mild (3.32±1.58) and moderate (3.58±1.71) epilepsy, and healthy controls (Figure 1a). All statistical analyses were performed by using one-way ANOVA. After treatment with valproate acid, the mean plasma microRNA-134 level of patients with severe epilepsy was significantly down-regulated from 5.62±1.60 to 3.26±2.80 (*P*<0.05) (Figure 1b). All data analyses were performed with student's *t*-test.

**Figure 2:**
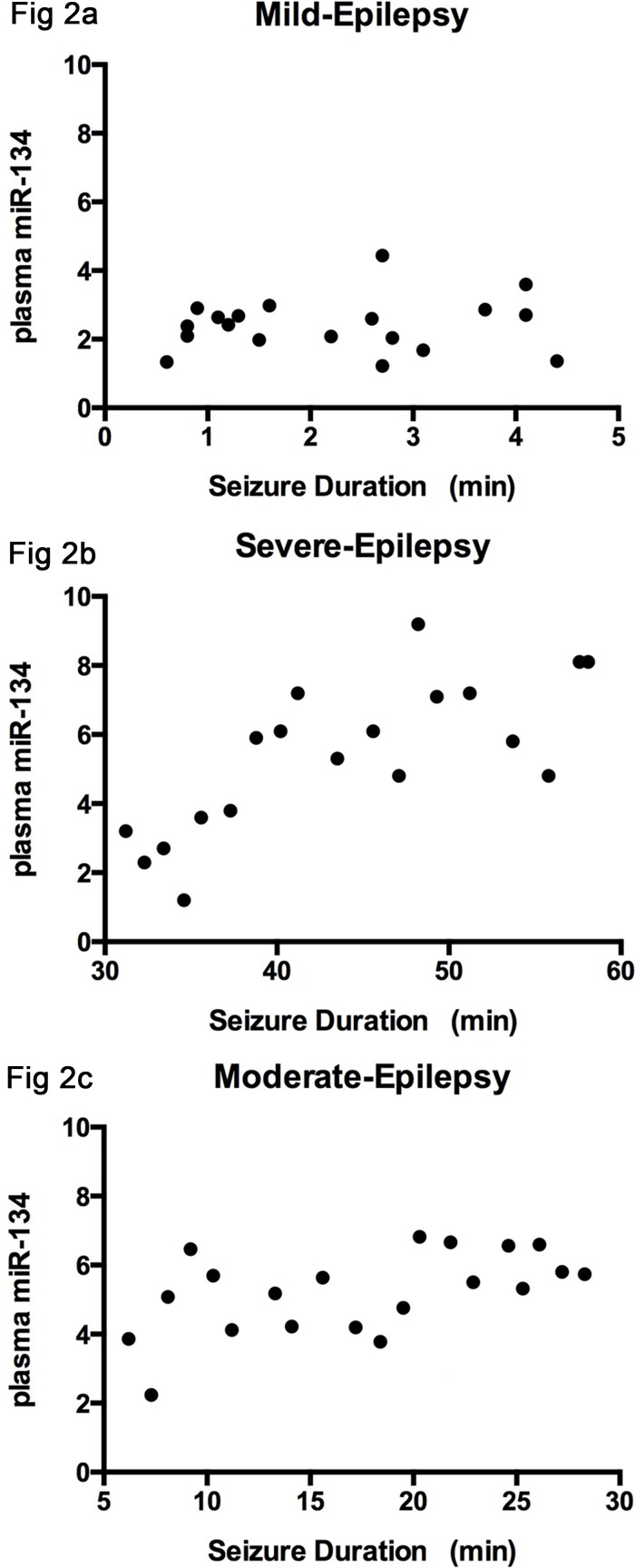
The correlation between the severity of seizure symptoms and plasma level of miRNA-134 There is no correlation observed in mild group (*r*=0.101), **(Figure 2a)**; A positive association was identified in the moderate and severe groups (*r*=0.533, *r*=0.746), as seen in **(Figure 2b** and **2c)**.

A significant positive correlation was observed between the severity of seizure symptoms and plasma miRNA levels in the moderate and severe seizure groups (*r*=0.533, *P*=0.016; *r*=0.746, *P*=0.003, respectively), whereas no correlation was observed in the mild seizure group (*r*=0.101, *P*=0.682) (Figure 2). All data analyses were calculated with Pearson's correlation test.

As shown in Table 1, clinical characteristics were assessed as continuous variables in 59 epilepsy patients and healthy controls. Among the continuous clinical variables shown in Table 1, except for the severity of seizure, none of the remaining continuous variables was associated with the plasma level of miRNA-134. Moreover, no significant correlation was noted between the plasma level of miRNA-134 and the aforementioned categorical variables.

**Table 1:** Characteristics of patients with epilepsy and health controls

Variable	Healthy controls	Mild epilepsy	Moderate epilepsy	Severe epilepsy
Age (yrs)	28.23±12.37	33.28±5.89	26.97±13.23	29.22±8.62
Gender (male: female)	9:11	13:7	8:12	10:9
Number of episode (Number/ month)	N/A	1.2±0.6	1.5±0.7	1.3±0.5
Mean duration of episode (Seconds)	N/A	2.22±1.22	17.35±6.96	43.93±8.56

## DISCUSSION

Although several studies have demonstrated the alteration of miRNA levels in human and animal models with temporal lobe epilepsy, the correlation between the expression of miRNAs and clinical characteristics of epilepsy has not been investigated [12, 13]. To our knowledge, this is the first study to analyze the association between the altered expression of circulating miRNAs and the duration of epileptic seizure. One major finding of this study was that plasma miRNA-134 level in the patients with severe epilepsy was significantly higher than that in healthy controls. This alteration may reflect the pathophysiological processes related to the neurobiology in patients with epilepsy, like an abnormal quantity or functioning of miR-134-associated silencing complex by unknown mechanisms, which plays a role in repressing Limk1 mRNA and several additional neuronal mRNAs translation. It limits the synthesis of new Limk1 and other neuronal proteins, and restricts the growth of dendritic spines [8], thereby leading to the disturbances of neural and synaptic plasticity which has been involved in the pathophysiology of epileptic disorders.

Moreover, the plasma level of miRNA-134 in the patients with severe epilepsy was significantly down-regulated at 1 month after valproate acid treatment, indicating that plasma miRNA-134 level was up-regulated in epilepsy patients, and the valproate acid probably led to the “normalization” of plasma miRNA-134 levels. These findings suggested that the changes in plasma level of miRNA-134 might be associated with the responses to the treatment of epilepsy. Thus, we speculate that the up-regulation of plasma miRNA-134 level probably serves as a biomarker in epileptic seizure and the down-regulation of plasma miRNA-134 levels possibly results from effective anti-epileptic treatment.

Our study also revealed significantly positive correlation between the plasma level of miRNA-134 and the seizure duration before treatment in epilepsy patients, suggesting that the presence of miR-134 in circulation may be accompanied by the progression of epileptic seizure. Accordingly, we postulate that miRNA-134 may be used as a correlation factor of the severity of epileptic seizure. Peng *et al*. reported the levels of miR-134 and 124 were up-regulated in both rat models and children with MTLE, and suggested that both can be potential targets of anticonvulsant drugs in the treatment of epilepsy [12]. Spain *et al*. also found that the level of miR134 was up-regulated in patients with epilepsy [13]. These results are consistent with the findings of this study. However, previous studies fail to explore the relationship between the expression of miR134 and clinical characteristics of epilepsy.

The miR-134 regulates dendritic spine development though a mRNA encoding a protein kinase, Limk1, that controls synaptic development, maturation and/or plasticity [8]. A unique feature of the miR-134-containing complex/Limk1 mRNA interaction is its reversibility: Brain-derived neurotrophic factor (BDNF), a neurotrophin released in response to synaptic stimulation, can release the inhibition on Limk1 translation imposed by mir-134 [8]. It seems that miRNA-134 and BDNF did opposite actions in the brain in regulating dendritic spine development. Previous studies have demonstrated that BDNF levels are up-regulated in hippocampus of rat models with epilepsy, which can be down-regulated by delivery of VPT therapy [14, 15]. We speculate that the down-regulated level of BDNF after valproate acid treatment may inhibit the binding of Limk1 mRNA and miR-134-containing complex, so the dissociative miR-134 may be up-regulated, and the miR-134-bound complex may be down-regulated in the brain. The miR-134 may dissociate from the Limk1 mRNA upon BDNF up-regulation, probably associated with the restoration of plasma miRNA-134 levels. Further studies designed to clarify the association between brain and circulating miRNA-134 levels are warranted.

Some limitations should be considered when interpreting the results of the study. First, our findings might have been strengthened with a larger number of study participants, particularly in regard to increasing the power of statistical analyses. Second, the lack of evidence measured plasma miRNA-134 correlated with miRNA-134 in the brain and the sources of plasma miRNA-134 are still unknown. The third is the definition of new-onset epilepsy. According to the most recent ILAE hypothesis [16], a seizure is a transient occurrence of signs and/or symptoms due to abnormal excessive or synchronous neuronal activity in the brain. Epilepsy was defined as “a disorder of the brain characterized by an enduring predisposition to generate epileptic seizures, and by the neurobiological, cognitive, psychological, and social consequences of this condition. The definition of epilepsy requires the occurrence of at least one epileptic seizure”. The author believes that we have not yet arrived at a final comprehensive definition of epilepsy as there is increasing evidence of complex changes in the neurobiological environment in the brain in which the single seizure only reflects one piece of the puzzle.

In conclusion, our findings indicate that plasma level of miR-134 at the onset of epilepsy may reflect the patho-physiological processes as well as the severity and duration of seizure. Plasma level of miRNA-134 in epilepsy probably serves as a potential peripheral marker that can respond to acute epileptic episode, which is associated with AEDs treatment. Whether it represents a pathologic or a clinical response remains to be determined. In future studies, plasma miRNA-134 levels should be measured in patients with various syndromes, such as GTCS and partial seizures to determine the specificity for this finding.

## MATERIALS AND METHODS

Epilepsy patients were recruited at Huaihua Hospital, China. Diagnosis of epileptic seizure was established according to the International League Against Epilepsy (ILAE) guidelines. The severity of epileptic seizure was categorized by a seizure severity questionnaire (SSQ), which has 16-item SSQ and each item is scored on a 7-point Liker scale, with lower scores representing lesser impact [severity: very mild (1) to very severe (7). Overall assessment of seizure severity is measured with the last two items (8 and 9), for which the total score ranges from 0 (minimum severity) to 7 (maximum severity) [17]. Patients with a score < 3 were allocated into the mild group, 3-5 into the moderate group and >5 into the severe group. The scale is based on an overall assessment of duration of seizure, seizure recovery time and loss of consciousness. Patients presenting with rapid cycling, mixed episodes, or severe or unstable clinical illnesses were excluded from subsequent study. Fifty-nine new-onset patients were enrolled in this study. The seizure data were collected during the video-EEG monitoring and one epileptologist blind to laboratory data analysis and calculation. Twenty-four patients were treated with phenytoin (250 mg daily) and 15 other patients were treated with gabapentin (250 mg daily) and 1 patient with carbamazepine (300 mg daily) for. Nineteen patients were only treated with valproate acid (300 mg daily) for 2 weeks. Patients did not receive any medication at 2 weeks before blood sampling to exclude the effect of AEDs on the expression of miRNA profiles. In the control group, 20 subjects without epileptic seizures confirmed by EEG were matched with epilepsy subjects by gender and age (28.23±12.37 years). The duration of EEG test was around 20-30 minutes. Baseline data including age, age at onset, number of seizure episodes, duration of seizure before blood sampling were collected. The expression of miRNAs was calculated utilizing the 2^−**ΔΔ**Ct^ method: ΔΔCt =[ΔCt miR-134 (calibrator sample)- ΔCt cel-lin-4 (calibrator sample)] - [ΔCt miR-134 (unknown sample) -ΔCt cel-lin-4 (unknown sample)]. Written informed consents were obtained from all participants. The study procedures were approved by the Institutional Review Board (IRB) of our hospital.

### Plasma isolation and preparation

The time interval between oral administration of VPA and plasma sampling was 1 h. For miRNA detection, whole blood (WB) samples (2.5 mL per patient) were collected from the subjects via a direct venous puncture into tubes containing sodium citrate, centrifuged at 2000 *g* for 5 min, and then the supernatant (plasma) was carefully transferred into an RNase-free tube for RNA extraction. The resultant plasma was aliquoted into eppendorf tubes and stored at −80°C.

### RNA extraction

A portion of 50 ml plasma was incubated at 56°C for 1.5 h with 0.65 mg/ml Proteinase K (Genview, 20 mg/ml). Synthetic cel-lin-4 (Shanghai GenePharma) was spiked-in as controls before acid phenol: chloroform extraction and then RNA was ETOH precipitated on at 20°C. Next, DNase treatment was performed to eliminate residual DNA fragments. Finally, after a second acid phenol: chloroform extraction, the pellet was re-suspended in DDW and synthetic cel-lin-4 was spiked-in as controls.

### qRT-PCR

RT primers were RNA was incubated in the presence of poly (A) polymerase (PAP; Takara-2180A), MnCl_2_, and ATP for 1 h at 37°C. Then, using an oligodT primer harboring a consensus sequence, reverse transcription was performed on total RNA using SuperScript II RT (Invitrogen). Next, the cDNA was amplified by real-time PCR; this reaction contained a miRNA specific forward primer, a TaqMan probe complementary to the 40 of the specific miRNA sequence as well as to part of the polyA adaptor sequence, and a universal reverse primer complementary to the consensus 40 sequence of the oligodT tail [17]. RNU19 was used for normalization. For each PCR, 5 μL template cDNA, equivalent to approximately 100 pg total RNA, was mixed with 10 μL×2 SYBR Green PCR master mix and 5 pmol each of the forward and reverse primers in a final volume of 20 mL. The amplification procedures included 15 s at 95°C and 1 min at 60°C for 45 cycles, and followed by thermal denaturing step to generate the dissociation curves. This standard PCR protocol was used for quantifying transcript levels of miRNAs differing by two or more nucleotide sequences. All reactions were run in triplicate. PCR product sizes were validated by electrophoresis using 2% agarose or 12% poly- acrylamide, 8 M urea gel. The RT primers used were (1) hsa-mir-134: CTCAACTGGTGTCGTGGAGTCGGCAATTCAGTTGAGCCCCTCTG, (2)hsa-mir-134 forward: ACACTCCAGCTGGGTGTGACTGGTTGACCA- GAGG, (3)hsa-mir-134 reversed: CTCAACTGGTGTCGTGGA.

### Statistical analysis

Most of the miRNA values fitted to a standard distribution curve and were therefore subjected to parametric analyses. Data analysis was performed through the SPSS for Windows (16.0, SPSS Inc., Chicago, Illinois). All the values are presented as mean ± standard deviation (M±SD). *P* values <0.05 were considered statistically significant. Continuous variables were compared using analysis of variance (ANOVA) and t test, as appropriate. Furthermore, *t*-test was used to compare miroRNA-134 measurements with each categorical clinical feature as the independent variable. Pearson's correlations were used to examine the relationship of continuous variables to plasma miRNA-134 level.

## Abbreviations

TLE: Temporal lobe epilepsy
miRNAs: MicroRNAs
VPA: Valproic acid
AEDs: anti-epilepsy drugs
LIMK1: LIM kinase-1
WB: whole blood
BDNF: Brain-derived neurotrophic factor
